# Validity and Sensitivity to Change of a Self‐Report Quality of Life Measure in Patients With Korsakoff's Syndrome

**DOI:** 10.1002/gps.70103

**Published:** 2025-05-21

**Authors:** Yvonne C. M. Rensen, Corrie de Waal ‐ Gordijn, Roy P. C. Kessels

**Affiliations:** ^1^ Vincent van Gogh Institute for Psychiatry Centre of Excellence for Korsakoff and Alcohol‐Related Cognitive Disorders Venray the Netherlands; ^2^ Radboud University Donders Institute for Brain Nijmegen the Netherlands; ^3^ Salios Regional Expertise Centre Korsakoff Het Dijckhuis Dordrecht the Netherlands; ^4^ Tactus Addiction Care Deventer the Netherlands

**Keywords:** alcohol use disorder, autonomy, feeling at home, illness insight, korsakoff's syndrome, long‐term care, quality of life

## Abstract

**Background:**

Assessing (self‐reported) quality of life (QoL) in patients with Korsakoff's syndrome (KS) is important to gain insight into these patients' well‐being and to optimize their care in long‐term care facilities. In this study, we describe the development of the QUALIKO‐Self Report (QUALIKO‐SR), an instrument for objectifying self‐reported QoL in patients with KS. Next, we compared the QUALIKO‐SR scores with the scores on the QUALIKO‐Proxy Version (QUALIKO‐PV) and examined changes in QoL over time. Finally, we assessed the convergent validity and investigated whether QUALIKO‐SR scores were related to the severity of the cognitive impairments.

**Methods:**

The study took place in specialized long‐term care facilities providing care for patients with KS. 116 patients with alcoholic KS participated in this study. The QUALIKO‐SR was developed and validated against the QUALIKO‐PV, the Manchester Short Assessment of Quality of Life (MANSA‐16), and the Montreal Cognitive Assessment 8.1 (MoCA).

**Results:**

Significant differences were found between self‐ and proxy reported QoL on the subscales Negative Affect, Social Isolation, and Feeling at Home. No significant differences were found on the other subscales. QUALIKO‐SR scores did not significantly vary over time. However, caregivers reported significant improvements in Care Relationships, Autonomy, Restless Tense Behavior, Social Isolation, and Feeling at Home over time. A significant, positive association was found between the QUALIKO‐SR and the MANSA‐16. No significant correlations were found between the QUALIKO‐SR and the MoCA.

**Conclusions:**

This study describes the development and validation of a self‐report instrument for objectifying QoL in patients with KS living in 24‐h care facilities, the QUALIKO‐SR. Measuring QoL in patients with severe cognitive impairments, such as patients with KS, is complex and we advise to include both self‐report and proxy‐report measures in future studies as well as in clinical practice. The availability of the QUALIKO‐SR and QUALIKO‐PV encourages researchers and clinicians to do so in patients with KS.

**Summary**

- A novel instrument, the QUALIKO‐Self Report (QUALIKO‐SR) is introduced to objectify self‐reported quality of life (QoL) in patients with Korsakoff's syndrome (KS).
- The QUALIKO‐SR correlated positively with the MANSA‐16, a self‐report measure of QoL used in psychiatric settings, indicating a good convergent validity, but did not correlate with the Montreal Cognitive Assessment as an index of cognitive status.
- The QUALIKO‐Proxy Version ratings by their caregivers did not differ from the QUALIKO‐SR ratings on most subscales, indicating that patients with KS can validly self‐report their QoL in the here‐and‐now, despite often limited illness insight and severe cognitive disorders.

## 1 Introduction

Korsakoff's syndrome (KS) is a neuropsychiatric disorder resulting from thiamine deficiency, typically occurring in the context of chronic, excessive alcohol use. The syndrome is characterized by disproportionate learning and memory impairments compared to other cognitive domains. In addition, neuropsychiatric symptoms, such as lack of illness insight, apathy, and confabulations are often present [1, 2]. Due to these severe cognitive impairments and neuropsychiatric symptoms, most patients with KS are in need of lifelong specialized care. As the incidence of KS is the highest in the age group of 50–59 [3], many patients require long‐term care for a substantial part of their lives. In the Netherlands, patients often reside in long‐term care facilities that are part of specialized nursing homes.

Patients with KS can become frustrated in long‐term care settings, as they may not fully comprehend why they are unable to live independently. Behavioral symptoms, such as agitation, aggression, and depressive disorders are highly prevalent in patients with KS living in 24‐h care facilities [4]. Assessing quality of life (QoL) in patients with KS is important to gain insight into these patients' well‐being and to optimize their care. Widely used health‐related QoL instruments include the World Health Organization Quality of Life ‐ Brief version [5], the 36‐Item Short‐Form Health Survey (SF‐36) [6], the 12‐Item Short‐Form Health Survey (SF‐12) [7]. Another widely used instrument is the Manchester Short Assessment of Quality of Life (MANSA) [8] which was developed for application in individuals with (severe) mental illness [9, 10]. However, none of these instruments have been studied in patients with KS. A key limitation of the use of these health‐related QoL instruments in patients with KS living in long‐term care facilities is their failure to account for this specific context. To accurately assess the QoL of individuals residing in long‐term care settings, it is essential to address issues like the (potential) lack of autonomy, feelings of being at home, and the quality of interactions and relationships with healthcare professionals and other residents. Informant‐based instruments developed to assess QoL in patients living in long‐term care facilities largely focus on patients with dementia. The QUALIDEM [11] and the Dementia Quality of Life (DEMQoL) [12] scale are recommended for use in older people with cognitive impairment living in long‐term care facilities. Although the QUALIDEM has several useful scales for patients with KS, this instrument is not tailored to this patient group. Patients with KS are younger and more active than patients with dementia. Relevant dimensions, such as ‘Meaningful activity’ (focusing on work‐related activities) and ‘Autonomy’ are lacking in the QUALIDEM. In addition, several dementia‐related items are not particularly relevant (e.g. “Cries”, “Calls out”). Patients with KS are responsive and alert; dementia‐related symptoms such as vocalization, wandering and aberrant motor behavior [13] – which are included in dementia‐specific QoL instruments – are uncommon in patients with KS.

Despite these limitations, previous studies [14, 15, 16] used the QUALIDEM to assess QoL in patients with KS, as Korsakoff‐specific instruments were not available at the time. Also, the QUALIDEM is an informant‐based QoL rating scale, administered in proxies (such as family members or nursing staff) who are asked to provide information on the patient's functioning and wellbeing. This approach overcomes the severe cognitive impairment and lack of illness insight [17, 18] that may hamper the validity of self‐report measures in patients with KS. One study, using the QUALIDEM showed that patients with KS living in long‐term care facilities tend to feel less at home than patients with dementia [14]. Furthermore, the QoL scores of KS patients on the QUALIDEM were found to remain relatively stable over a 20‐month period [15]. Moreover, QoL of patients with KS in long‐term care facilities improved after an intervention teaching patients meaningful activities using an errorless learning approach [16].

Recently, the QUALIKO was developed to better capture the QoL in patients with KS, with promising measurement qualities [19]. The QUALIKO is a feasible observation scale i.e. based on the QUALIDEM, but with some important, Korsakoff‐specific changes, such as two additional dimensions of QoL that are relevant for this population (i.e. autonomy and meaningful activities). Caregivers or relatives who know the patient well are asked to complete the QUALIKO to quantify the patient's QoL. Even though proxy assessments may have added value, especially when patients are unable to reflect upon their current situation, they never fully capture the patient's own perception of their position in life [20]. Even though patients with KS have severe cognitive impairment and lack of illness insight, they may still be able to complete self‐report questionnaires and reflect on how their feeling in the present moment. A self‐report instrument to assess QoL in patients with KS could thus be an important addition to the recently developed observation scale.

The purpose of this study was to develop and validate a self‐report version of the QUALIKO for assessing self‐reported QoL in patients with KS living in 24‐h care facilities. Comparing the results of the self‐ and proxy reported QoL might provide important clinical insights. It may also serve as a feasible, standardized way to monitor self‐reported QoL over time, even in patients with severe cognitive deficits. In this paper we first describe the development of this self‐report scale. Next, the aims of this paper were: (1) to compare the scores of the patients with KS on the QUALIKO‐SR with the scores on the QUALIKO‐PV, (2) to examine changes in QoL over time, (3) to validate the QUALIKO‐SR against a widely used self‐report instrument to measure QoL in psychiatric patients, the MANSA‐16, and (4) to examine whether the QoL scores (self‐ and proxy reported) were related to the severity of the cognitive impairment.

## 2 Methods

### 2.1 Scale Development

A focus group consisting of health care professionals (nursing staff, residential counselor, psychologist, senior policy officer) from Korsakoff expertise center Salios adapted the proxy version of the QUALIKO to a self‐report version. The focus group assessed the items in the proxy‐version on the applicability in patients with KS (i.e., are the question formulated in an understandable way, is answering the questionnaire not too burdensome) and proposed relevant changes. Where possible, the same words and phrasings of the questions as in the proxy‐version have been used. Questions were rephrased (e.g., “Is in a good mood” [proxy version] was changed to “Are you in a good mood? [self‐report version])”, simplified or changed to best capture the perspective of the patient (e.g., “makes restless movements” [proxy version] was changed to “Are you feeling restless?” [self‐report version]). The items from subscales were clustered (e.g., the first seven questions of the self‐report version all belong to the subscale *Care Relationships*). Subsequently, the self‐report questionnaire was completed individually by all members of the focus group, with the aim of assessing whether the questions would be understood by the patient. The response was unanimously affirmative, leading to the inclusion of the revised questions in the final instrument. The QUALIKO‐SR consists of 41 items. The QUALIKO‐SR was piloted in 3 patients. The patients indicated that they understood and could answer the questions well. The patients also mentioned that they appreciated being asked about their opinions. Based on the pilot outcome, no changes were needed to the instrument.

The QUALIKO‐SR consists of the same 10 subscales as the proxy‐version: Care Relationships (7 items), Autonomy (4 items), Positive Affect (7 items), Negative Affect (2 items), Restless Tense Behavior (2 items), Positive Self‐image (2 items), Social Relationships (4 items), Social Isolation (3 items), Feeling at Home (4 items) and Meaningful Activities (6 items). The response options are, as in the proxy version, on a four‐point scale (from 0 = never, to 3 = frequently), with higher scores indicating a better QoL.

### 2.2 Scale Validation

#### 2.2.1 Participants

In order to detect a medium to large correlation between the QUALIKO‐SR and the MANSA‐16 (*r* > 0.3) [21], with a power (1‐β) of 0.9 and *α* = 0.05, a minimum sample size of 109 was recommended (G*Power 3.1.9.2). Patients were recruited from three long‐term care facilities in the Netherlands with a regional expertise center (REC) status (Salios in Dordrecht; ZorgAccent in Hellendoorn; and Magenta Zorg in Bergen), and one regular nursing home (MijZo in Waalwijk), all having specialized units exclusively for patients with KS. The long‐term care facilities cooperate in a Dutch network for innovative Korsakoff care. To be eligible for inclusion, patients had to meet the DSM‐5 [22] criteria for Alcohol‐induced Major Neurocognitive Disorder, amnesic‐confabulatory type (291.1, i.e., Korsakoff's syndrome), verified by neuropsychological assessment. In addition, the criteria for alcoholic KS had to be met [23]; that is, all were patients with a diagnosis of alcoholic KS who had a history of malnutrition or thiamine deficit (with evidence of a Wernicke encephalopathy), and a disproportionate memory disorder compared to all other cognitive domains. The impairments were not attributable to another medical condition or use of other substances, and none of the participants met the criteria for alcohol‐related dementia [24]. Participants had to have a verified history of excessive, chronic alcohol use. Exclusion criteria for this study were the presence of active psychiatric disorders that might interfere with cognitive functioning (e.g. major depressive disorder, schizophrenia). All patients had to be at least 6 weeks abstinent from alcohol. The level of formal education was assessed using a scale with seven categories based on the Dutch educational system, 1 being the lowest (less than primary education; i.e., six or fewer years of education) and 7 the highest (academic degree; i.e., 18 or more years of education). The data included in this manuscript were obtained in compliance with the Helsinki Declaration. Prior to the study, patients gave their written informed consent and this study was approved by the Institutional Review Board of Vincent van Gogh Institute for Psychiatry (CWOP# 2022‐JE/hr/007).

#### 2.2.2 Instruments

The QUALIKO‐PV is an observation scale, consisting of 42 items describing observable behaviors across 10 aspects of QoL relevant to patients with KS living in long term care facilities, that is, Care Relationships, Autonomy, Positive Affect, Negative Affect, Restless Tense Behavior, Positive Self‐image, Social Relations, Social Isolation, Feeling at Home, and Meaningful Activities [19]. All subscales, except for Negative Affect and Positive Self‐image, demonstrated acceptable to good internal consistency reliability and inter‐observer reliability was fair to excellent for all subscales. The QUALIKO total score (ranging from 0–126), the global QoL score (ranging from 1 – 10), and the subscale scores were used in this study, with a higher score indicating better QoL.

The MANSA [8] is a self‐report questionnaire, based on Lehman's [25] conceptualization of QoL and explores satisfaction with a number of life domains. It asks patients to rate how satisfied they are with several aspects of their lives, including finance, friendships and physical health. In this paper, the Dutch version of the MANSA‐16 that is recommended for use in routine outcome measurement in the Netherlands was used [26, 27]. It takes 5 – 10 min to complete the MANSA‐16 and the total score (the total score ranged from 0 = very poor QoL to 112 = the best QoL possible) was used as an outcome measure.

The Montreal Cognitive Assessment (MoCA) [28] was administered to assess the patients' current global cognitive functioning. The 8.1 version was administered and the total score (uncorrected for education) and memory index score (MIS) were used in the current study. The MoCA is widely used as a cognitive measure in patients with alcohol use disorder, as well as patients with KS [29, 30]. The MoCA was not used to classify individuals with and without cognitive impairments (as all patients are expected to perform in the impaired range due to their diagnosis).

#### 2.2.3 Procedure

Patients completed the QUALIKO‐SR (with the possibility of getting help form their primary professional caregivers). Primary professional caregivers, who knew the patients well, completed the QUALIKO‐PV and the MANSA‐16 within a time span of two weeks. All primary professional caregivers received a brief verbal and written instruction from the researchers on how to use the instrument. Primary professional caregivers were selected as the proxy raters, as patients with KS often no longer have—or have strained—relationships with family and/or friends, because of the history with addiction and the associated problems. Moreover, primary professional caregivers interact with the patient on a daily basis and have a good understanding of the patient's situation over the past week. The MoCA was administered as a cognitive screen by a trained psychologist or a psychology technician within 3 months after the completion of the other instruments. In a subsample of the patients, a follow‐up (consisting of the QUALIKO‐SR and ‐PV) was completed after approximately 5 months. Completion of the QUALIKO‐SR took approximately 20 min.

### 2.3 Analyses

#### 2.3.1 Missing Data and Assumptions Check

The data set was checked for missing item responses and missing data analyses were performed using Little's Missing Completely At Random (MCAR) test. To assess the normality of the data, we visually inspected the distribution using histograms and Q‐Q plots. The Shapiro‐Wilk test indicated a significant deviation from normality (*p* < 0.05). As a result, non‐parametric statistical tests were used for all analyses.

#### 2.3.2 Differences Between Self‐Reported and Proxy‐Reported QoL

Wilcoxon signed‐rank tests were conducted to examine the difference between patients self‐reported QoL and proxy‐reported QoL on the QUALIKO.

#### 2.3.3 Changes in Quality of Life Over Time

Wilcoxon signed‐rank tests were conducted to examine the difference between patients self‐reported QoL and proxy‐reported QoL on the QUALIKO over time.

#### 2.3.4 Relation Between QUALIKO‐SR and MANSA

Spearman ρ correlation coefficients were computed to assess the association between self‐reported QoL on the QUALIKO (total score) and the MANSA‐16 (total score).

#### 2.3.5 Relation Between Quality of Life and Cognitive Functioning

Spearman ρ correlation coefficients were computed to assess the relationship between self‐reported QoL on the QUALIKO (total score) and the MoCA 8.1 total score and MoCA Memory Index Score (MoCA MIS).

Effect sizes were computed for the Wilcoxon signed‐rank tests $\left(r=Z/\sqrt{n}\;\right)$. All analyses were performed using IBM SPSS Version 29.

## 3 Results

### 3.1 Demographics

A total of 116 patients with alcoholic KS living in specialized 24‐h care facilities participated in this study (86 men). The mean age was 65.8 years old (rage 41 – 81 years). Patients had a median educational level of 4, which is comparable to 9 – 11 years of education in the Anglo‐Saxon educational system. On average, they had been living in these facilities for 6.5 years (range = 2 weeks to 28 years) at the time of the assessment.

Table 1 shows the mean scores and standard deviation on the QUALIKO subscales reported by patients with KS (self‐report, *n* = 115) and their caregivers (proxy‐report, *n* = 116). Missing item responses on the QUALIKO‐SR ranged from 0.9% to 6.9%), which included one patient who did not complete the QUALIKO‐SR at all. Little's MCAR test showed that these missing items were not completely at random (χ^2^(457) = 516.9, *p* = 0.027). Visual inspection of the *n* = 115 responders showed that most missing item responses (4‐7) were from the Productivity subscale, as some patients did not have any work‐related activities and thus felt unable to complete these items. Missing item responses only occurred on 7 QUALIKO‐PV items (0.9%–2.6% missing item responses); Little's MCAR test showed that these missing items were completely at random (χ^2^(200) = 187.3, *p* = 0.731). Missing data were not imputed; if items were missing from one subscale, that subscale score was not computed for that case and no total score was computed, but the remaining subscales were included in the analyses.

**TABLE 1:** Mean scores and standard deviation (in parenthesis) on the QUALIKO subscales reported by patients with Korsakoff's syndrome (self‐report, *n* = 115) and their caregivers (proxy‐report, *n* = 116), ranging from 0 to 3, with higher scores indicating better quality of life.

	Self‐report	Proxy‐report	*p*‐value
Care relationship	2.1 (0.5)	2.1 (0.6)	0.456
Autonomy	2.1 (0.5)	2.3 (0.7)	**0.005**
Positive affect	2.5 (0.5)	2.6 (0.5)	0.448
Negative affect	2.1 (0.8)	2.3 (0.8)	**0.004**
Restless tense behavior	1.9 (0.9)	2.0 (0.9)	0.371
Positive self‐image	2.6 (0.6)	2.6 (0.7)	0.352
Social relations	2.2 (0.7)	2.1 (0.7)	0.250
Social isolation	1.5 (0.6)	2.0 (0.7)	**<** **0.001**
Feeling at home	2.0 (0.8)	2.3 (0.7)	**<** **0.001**
Meaningful activities	1.9 (1.0)	1.8 (0.9)	0.905

#### 3.1.1 Differences Between Self‐Reported and Proxy‐Reported QoL

Results from the Wilcoxon signed‐rank tests indicated that there were significant differences between self‐ and proxy reported scores on the subscales Autonomy (*n* = 112, *z* = −2.83, *p* = < 0.005, *r* = −0.27), Negative Affect (*n* = 115, *z* = −2.84, *p* = < 0.004, *r* = −0.26), Social Isolation (*n* = 114, *z* = −4.87, *p* = < 0.001, *r* = −0.46), and Feeling at Home (*n* = 112, *z* = −3.7, *p* = < 0.001, *r* = −0.35) (see Table 1). No significant differences were found on the subscales Care Relationships, Positive Affect, Restless Tense Behavior, Positive Self‐image, Social Relations, and Meaningful Activities (see Table 1).

#### 3.1.2 Changes in Quality of Life Over Time

The results over time for the subsample of 58 patients and their caregivers are presented in Table 2. Mean time difference between T1 and T2 was 5 months (SD = 5.7; the median was also 5 months). There were no significant changes in QUALIKO‐SR scores over time. However, proxies reported significant, positive changes in Care Relationships (all *p*‐values < 0.111) (*n* = 58, *z* = −3.1 *p* = 0.002, *r* = −0.41), Autonomy (*n* = 57, *z* = −4.2, *p* = < 0.001, *r* = −0.59), Restless Tense Behavior (*n* = 57, *z* = −2.50, *p* = 0.012, *r* = −0.33), Social Isolation (*n* = 58, *z* = −2.27, *p* = 0.024, *r* = −0.30), and Feeling at Home (*n* = 57, *z* = −3.1, *p* = 0.002, *r* = −0.41).

**TABLE 2:** Mean scores and standard deviation (in parenthesis) on the QUALIKO subscales reported by patients with Korsakoff's syndrome (self‐report) and their caregivers (proxy‐report), ranging from 0 to 3 over time with higher scores indicating better QoL (median = 5 months between T1 and T2).

	Self‐report			Proxy‐report		
	T1	T2	*p*‐value	T1	T2	*p*‐value
Global QoL rating*	7.3 (1.5)	7.0 (1.5)	0.581	6.9 (1.0)	7.3 (1.1)	0.024
Care relationship	2.3 (0.5)	2.3 (0.6)	0.857	2.0 (0.6)	2.3 (0.6)	**0.002** *
Autonomy	2.2 (0.5)	2.2 (0.6)	0.380	2.1 (0.7)	2.5 (0.7)	**<** **0.001** *
Positive affect	2.6 (0.5)	2.5 (0.6)	0.342	2.6 (0.4)	2.6 (0.5)	0.170
Negative affect	2.4 (0.7)	2.2 (0.8)	0.208	2.3 (0.8)	2.4 (0.8)	0.247
Restless tense behavior	2.2 (0.8)	2.2 (0.8)	0.801	2.0 (0.9)	2.2 (0.8)	0.012*
Positive self‐image	2.7 (0.7)	2.7 (0.6)	0.580	2.5 (0.8)	2.5 (0.8)	0.238
Social relations	2.3 (0.7)	2.3 (0.8)	0.747	2.2 (0.6)	2.3 (0.7)	0.360
Social isolation	1.5 (0.7)	1.4 (0.6)	0.748	2.0 (0.7)	2.2 (0.7)	**0.024** *
Feeling at home	2.0 (0.9)	2.1 (0.9)	0.464	2.2 (0.7)	2.6 (1.1)	**0.002** *
Meaningful activities	2.1 (0.8)	2.0 (0.8)	0.360	2.0 (0.8)	2.0 (0.9)	0.744

*Note:* this data represents a subsample (*n* = 58) of the total sample included in this study.

Abbreviation: QoL = Quality of Life.

* ranging from 1 to 10.

#### 3.1.3 Relation Between QUALIKO‐SR and MANSA

A significant, positive association was found between the total score of the QUALIKO‐SR and the total score on the MANSA‐16 (ρ(104) = 0.488, *p* = < 0.001).

#### 3.1.4 Relation Between Quality of Life and Cognitive Functioning

The MoCA was not administered in 36 patients due to logistic reasons. All patients in whom the MoCA was administered (*n* = 82) performed in the impaired range on the MoCA (mean total score 18.6, SD = 4.0; range 6–24; mean MoCA MIS 5.2, SD = 3.1) [31]. There were no significant correlations between the total score of the QUALIKO‐SR and the total score on the MoCA (all *p*‐values < 0.111) or the MoCA MIS (all *p*‐values > 0.341).

## 4 Discussion

In this paper we describe the development and validation of a self‐report instrument for measuring QoL in patients with KS living in 24‐h care facilities: the QUALIKO‐SR. This instrument is based on a validated proxy‐based instrument for measuring QoL in this population, the QUALIKO‐PV [19]. We will discuss the results comparing the self‐reported and proxy‐reported QoL, and changes in QoL in patients with KS living in long‐term care facilities over time. In addition we discuss the results regarding the validity of the QUALIKO‐SR, the relation between QoL and severity of cognitive impairment.

First, we showed that almost no missing item responses were present on the QUALIKO‐PV. In the QUALIKO‐SR, some patients felt unable to complete the items of the Meaningful Activities subscale, as they did not have any work‐like activities or activities they deemed meaningful. Only one patient did not complete the QUALIKO‐SR at all. These findings illustrate that both the QUALIKO‐PV and the QUALIKO‐SR are feasible measures that can be easily incorporated in daily practice for most patients and nearly all professional caregivers.

Next, self‐reported QoL of patients with KS living in long‐term care facilities was compared with proxy‐reported QoL. The ratings of patients and their caregivers did not differ on 6 of the 10 subscales. This indicates that the caregivers know the patients well, and are generally able to assess how the patient is doing. Caregivers gave higher (i.e., more positive) ratings on the subscales Autonomy, Negative Affect (indicating more positive affect), Social Isolation (indicating less social isolation), and Feeling at Home than the patients themselves. This more positive response tendency by proxies compared to the patients' self‐report ratings differs from results obtained in patients with dementia. In patients with dementia, differences between self‐reported and proxy‐reported (family and caregivers) QoL were found, but self‐reported QoL ratings were higher than the proxy‐reported QoL ratings [32, 33]. A systematic review on proxy‐rated QoL in patients with dementia showed that the raters' own levels of stress and burnout, experiencing the workplace as stressful and overwhelming, and experiencing challenging and agitated behavior by the patients adversely affected QoL scores [34]. The patients and raters in this study were recruited from expertise centers specialized in the care for patients with KS. This makes the raters particularly well equipped to provide care for these patients, possibly reducing caregiver burden compared to professional caregivers in less specialized centers. Moreover, our findings show a lot more concordance between views on QoL between the patients and caregivers included in this study, compared to a previous study comparing the reports of QoL from patients with alcohol‐related brain damage and their healthcare professionals [35]. The use of the Korsakoff‐specific QUALIKO, rather than a more general QoL instrument, might account for this. Future studies are encouraged to include rater‐specific factors when assessing QoL in patients with KS using proxy‐reports by healthcare professionals. The results of the current study suggest that increasing autonomy, reducing negative affect, social isolation, and (not) feeling at home might deserve extra attention from caregivers, as patients feel less positive about these topics than raters observed.

In a subsample, self and proxy‐reported changes in QoL over time were examined. Caregivers reported improvements in Care relationship, Autonomy, Restless Tense Behavior, Social Isolation and Feeling at Home after 5 months. This is in accordance with previous findings who administered the QUALIDEM at baseline and after 20 months in patients with KS [15]. They also reported significantly higher scores on Care Relationships and Feeling at Home (note that the subscale Autonomy is not included in the QUALIDEM). The improvements in QoL can be explained by several factors. First, they may result from the specialized care provided by a multidisciplinary expert team in the expertise centers. In addition, some of the participating long‐term care facilities administered the QUALIKO‐SR and QUALIKO‐PV as part of the semi‐annual multidisciplinary treatment evaluations of the patients. Therefore, interventions might have been deployed based on the results of the QUALIKO, which might have improved aspects of QoL. Second, the largest differences in self‐report and proxy‐report where found on the subscales Autonomy, Social Isolation and Feeling at Home. These constructs might be more difficult to observe than, for example, Meaningful Activities or Restless and Tense Behavior. Last, due to the their memory impairments, it may also be difficult for patients to remember changes that are made in their daily lives or to update their thoughts and remember their feelings over the past week. Therefore, they might not notice the improvements that their caregivers do observe. The patients with KS in this study did not report any differences in QoL over time. This might also indicate that QoL is stable in patients with KS living in long‐term care facilities.

The QUALIKO‐SR was validated against a widely used self‐report instrument to measure QoL in psychiatric patients, the MANSA‐16. A significant, positive association was found, establishing the convergent validity of the QUALIKO‐SR. The concept op QoL of the MANSA‐16 is not specific to health or disease related issues. As a result, it can be used to compare patients with different types of psychiatric diagnoses [36]. However, for examining QoL in patient with KS in more depth, a (more extensive) disease specific instrument, such as the QUALIKO can be of great value. It can be easily incorporated in daily practice as a monitoring instrument, particularly to support the evaluation of treatment plans. For example, if a patient with KS rates their relationship with healthcare staff significantly lower than the professionals themselves, this discrepancy can highlight a need for targeted interventions. In contrast, generic instruments such as the MANSA provide a general overview of an individual's self‐reported quality of life, but remain broad and do not account for contextual factors, such as residing in a long‐term care facility. Therefore, the QUALIKO offers a more precise and context‐sensitive approach to assessing and improving patient well‐being.

Lastly, the QoL scores (self‐ and proxy reported) were unrelated to the severity of the cognitive impairment. Because of the profound effects of the cognitive impairments on daily life, it may be expected that QoL would be lower in patients with more severe cognitive impairments. For example, is has been demonstrated that prospective memory difficulties adversely impacted QoL in community‐dwelling older adults who experienced problems managing their instrumental activities of daily living [37]. However, lack of illness insight may affect self‐reported QoL more so than the severity of cognitive impairments. In patients with mild cognitive impairment or Alzheimer's disease, individuals who were unaware of their diagnosis reported higher QoL than patients who showed more illness insight. This relationship was unrelated to the severity of cognitive impairment [38]. Recently, the significant relation between a greater lack of illness insight and better QoL has also been found in patients with KS. A network analysis study showed that impaired awareness was most strongly associated with QoL in patients with KS living in long‐term care facilities. Patients who are less aware of their situation tend to experience better QoL [39].

A limitation of this study is that, in addition to cognitive impairments, neuropsychiatric symptoms are frequently present in patients with KS. However, we did not include instruments to examine the relationship between QoL and the severity of neuropsychiatric symptoms, such as the Neuropsychiatric Inventory‐Questionnaire [40]. We recommend incorporating such measures in future research. In conclusion, this study describes the development and validation of a self‐report instrument for objectifying QoL in patients with KS living in 24‐h care facilities, the QUALIKO‐SR. This instrument can be used, next to the validated and feasible QUALIKO proxy‐report. Measuring QoL in patients with severe cognitive impairments, such as patients with KS, is complex and we advise to include both self‐report and proxy‐report measures in future studies as well as in clinical practice. The availability of the QUALIKO‐SR and QUALIKO‐PV encourages researchers and clinicians to do so in patients with KS.

## Ethics Statement

All data used in this manuscript have been collected in accordance with the Declaration of Helsinki. Ethical approval has been obtained from the Institutional Review Board from Vincent van Gogh Institute for psychiatry (CWOP# 2022‐JE/hr/007). All files were stored and analyzed in a fully anonymous format compliant with the EU General Data Protection Regulation 2016/679.

## Consent

Written informed consent was obtained from all patients.

## Conflicts of Interest

The authors do not have any conflicts of interest to report.

## Data Availability

All data are available from the corresponding author upon request, as the informed consent forms completed by all participants did not ask for permission to share the data via a data repository. Further enquiries can be directed to the corresponding author.
